# Pancreaticoduodenectomy for trauma in an adolescent female with complex pancreatic and duodenal injuries: Case report and review of the literature

**DOI:** 10.1016/j.tcr.2024.101034

**Published:** 2024-05-09

**Authors:** Sarah E. Kim, Farah Al Rahmani, Rembrandt VanDruff, Marc Mesleh, J. Kayle Lee

**Affiliations:** a Department of Surgery, University of Illinois at Chicago, Chicago, IL, United States of America; b Advocate Christ Medical Center, Oak Lawn, IL, United States of America

**Keywords:** Trauma Whipple, Pancreaticoduodenectomy, Pancreatic injury, Adolescent, Blunt trauma

## Abstract

**Background:**

We present a successful staged surgical repair of an adolescent who sustained a high grade combined pancreaticoduodenal injury following a high-speed motor vehicle collision.

**Methods:**

We discuss our case as well as provide a thorough literature review made on databases such as PubMed, Google Scholar, and Embase.

**Summary:**

A fifteen-year-old female presented after a motor vehicle collision with abdominal pain and imaging suggestive of pancreatic and duodenal injuries. Emergent exploratory laparotomy confirmed a transection of the pancreatic neck in addition to disruption of the second portion of the duodenum. She sustained other injuries including an injury to the portal vein and a right colonic perforation. A damage control strategy was employed, and the patient underwent duodenal repair, wide drainage of the pancreatic injury, primary portal vein repair, right hemicolectomy, and temporary abdominal closure using negative pressure wound dressing placement. She remained stable overnight in the ICU and was taken back to the operating room for a pylorus-preserving pancreaticoduodenectomy with a hepatobiliary surgeon the following afternoon. The patient required additional surgery for fixation of an unstable vertebral fracture but was discharged to inpatient rehab within two weeks of presentation. She did not require TPN, and the only long-term sequelae have been admissions for acute uncomplicated pancreatitis that have been treated medically.

**Conclusion:**

Combined pancreatic and duodenal injury in the pediatric population is uncommon. We discuss our case of a patient requiring a pancreaticoduodenectomy. Despite postoperative pancreatitis and limited information in this field, we believe we provided the optimal surgical care, and this is a potential area for future investigation.

## Introduction

Combined severe pancreatic and duodenal injuries in the pediatric population are rare, accounting for only 0.3% of all pediatric trauma [1]. Surgical options for management include immediate reconstruction, delayed reconstruction, and staged reconstruction. There is a lack of significant prospective or retrospective data to guide the surgeon's treatment of pediatric patients. This case represents a successful outcome using the recommendations for adult pancreatic and duodenal injuries: initial damage control exploratory laparotomy followed by early (<48 h) staged reconstruction by a surgeon with hepatopancreaticobiliary experience.

## Case description

Our case is of a 15-year-old female with no significant past medical history who presented to a Level I Trauma Center following a high-speed (>100 miles per hour), head-on motor vehicle collision (MVC) into a concrete wall as a restrained back seat passenger. On arrival, the patient was hemodynamically stable with a patent airway, non-labored breathing, and a Glasgow Coma Score of 15. Primary survey revealed a seatbelt sign across the chest and abdomen with noted abdominal tenderness to palpation on exam.

**Fig. 1:**
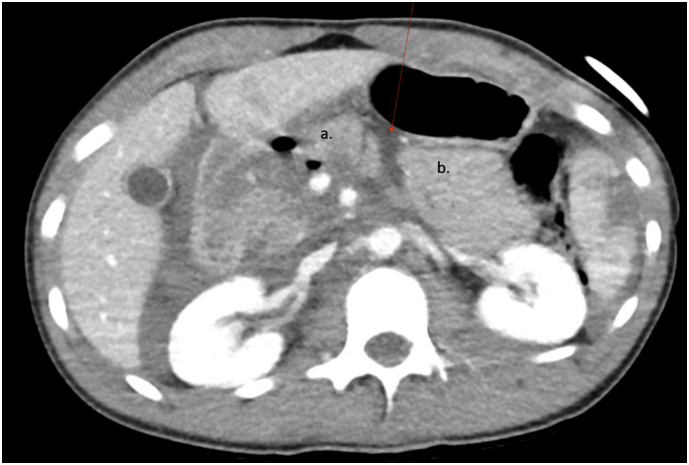
Region of complex intermediate density separating the (a) head and (b) body of the pancreas.

Plain radiography revealed 3 rib fractures on the left and widening of the left sacroiliac joint. The patient also underwent several computed tomography (CT) scans with findings including a large right retroperitoneal hematoma, foci of free air, a 3 cm Grade II left lobe hepatic laceration, and a 6 cm Grade III splenic laceration. A region of complex intermediate density fluid separating the head and body of the pancreas was concerning for transection (Fig. 1), and non-visualization of the ventral wall of the third portion of the duodenum was concerning for a duodenal injury. Other injuries included bilateral L1-L2 facet joint subluxation with widening of L1-L2 interspinous distance suggesting disruption of posterior ligaments and fractures of the left lateral ribs six through eight. Given CT findings and interval hematemesis, the patient was taken emergently to the operating room for an exploratory laparotomy.

**Fig. 2:**
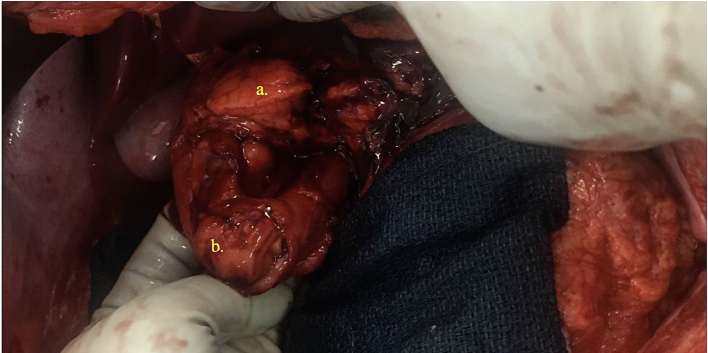
Complete avulsion of the pancreatic head and neck denoting a Grade V pancreatic injury and Grade IV injury to the second portion of the duodenum.

Surgical exploration was significant for a right retroperitoneal zone I hematoma, right colon serosal and mesenteric injuries, Grade II liver intraparenchymal hematoma, and superficial non-bleeding splenic laceration. There was also a Grade IV injury to the 2nd portion of the duodenum involving >75 % of the circumference with gross spillage of bile. The pancreatic uncinate process and body were transected (Fig. 2), consistent with a Grade V pancreatic injury (Table 1, Table 2). There were two bleeding branches of the pancreaticoduodenal artery that were ligated, and a posterior portal vein injury was primarily repaired. Given the extent of the pancreaticoduodenal injuries, it was decided to do damage control and utilize a staged approach to reconstruction. The duodenal defect was closed to control contamination, and Jackson-Pratt drains were left in the pancreatic bed and near the duodenal injury. The right colon was ischemic from the mesenteric injury and therefore resected. The patient was left in discontinuity and the abdomen was temporarily closed using a vacuum dressing. Estimated blood loss was approximately 800 mL, and 2 units of packed red blood cells were transfused intraoperatively.

**Table 1:** AAST Organ Injury Scale duodenal injury [4].

Grade	Type of injury	Description of injury
I	Hematoma	Involving single portion of duodenum
	Laceration	Partial thickness, no perforation
II	Hematoma	Involving more than one portion
	Laceration	Disruption <50 % of circumference
III	Laceration	Disruption 50–75 % of circumference of D2
		Disruption 50–100 % circumference of D1,D3,D4
IV	Laceration	Disruption >75 % of circumference of D2
		Involving ampulla or distal common bile duct
V	Laceration	Massive disruption of duodenopancreatic complex
	Vascular	Devascularization of duodenum

**Table 2:** AAST Organ Injury Scale pancreatic injury [4].

Grade	Type of injury	Description of injury
I	Hematoma	Minor contusion without duct injury
	Laceration	Superficial laceration without duct injury
II	Hematoma	Major contusion without duct injury or tissue loss
	Laceration	Major laceration without duct injury or tissue loss
III	Laceration	Distal transection or parenchymal injury with duct injury
IV	Laceration	Proximal transection or parenchymal injury involving ampulla
V	Laceration	Massive disruption of pancreatic head

In the ICU, patient remained hemodynamically stable, and mild metabolic acidosis corrected with fluid resuscitation. The following day the patient was taken back to the operating room with the trauma service and a hepatobiliary surgeon. Because the pancreatic head and the duodenum were avulsed from the body and tail, the decision was made to do a pylorus preserving Whipple procedure, a pancreaticoduodenectomy. A cholecystectomy, hepaticojejunostomy, pancreaticojejunostomy, and duodenojejunostomy were all performed in standard fashion. The Jackson-Pratt drains were repositioned near the anastomoses. Bowel continuity was restored with an ileocolic anastomosis, and the abdomen was irrigated and closed.

The patient's postoperative course was unremarkable, without any clinical signs of pancreatic leak or other complications. After surgical lumbar spine stabilization, the patient was discharged during the second week of admission. Over the course of the next 4 years the patient has had multiple readmissions for remnant pancreatitis which have been medically managed. Workup for pancreatic duct stricture has been negative, however genetic testing revealed pathogenic variants in the CTR and SPINK1 genes - this combination may cause a predisposition to pancreatitis.

## Etiology

The most common cause of pediatric pancreatic and duodenal injuries within the pediatric population is blunt trauma compared to penetrating trauma in adults [2]. A review of the National Trauma Data Bank from 2007 to 2011 and found the most common cause of high grade pancreatic injury to be MVC (55 %) followed by bicycle accidents (19.7 %) and then abuse (14.1 %) [1]. The mortality rate associated with combined pancreaticoduodenal is reported as high as 31–50 % in adults compared to 26.5 % in children [2,3].

## Diagnosis

Diagnosis is typically made with a CT with intravenous contrast if the patient is hemodynamically stable. Oral contrast has not been shown to improve sensitivity in detecting pancreatico/duodenal injuries. However, CT can appear normal in 20–40 % of patients presenting within 12 h of pancreatic injury. In the absence of definitive diagnosis, serum amylase and lipase levels obtained every 3–6 h can provide prognostic information [4]. In the stable patient, if CT is equivocal, urgent ERCP or dynamic secretin-stimulated MRCP can also aid in the diagnosis of pancreatic injury, and more specifically, involvement of the main pancreatic duct [2].

## Management

In the adult population, operative management is the standard for high grade pancreatic trauma. Most commonly pancreatic and duodenal injuries are classified using the grading system for solid and hollow organs originally published in the American Association for the Surgery of Trauma (AAST) in 1990 [5]. In setting of hemodynamically stable blunt grade I and II injuries, patients can be managed non-operatively. In penetrating injuries, non-operative management is not recommended, and grading is often established in the operating room. Any patient with hemodynamic instability, peritonitis, or evisceration should be taken to the operating room for exploration. Patients with lesions that require pancreaticoduodenectomy should initially undergo damage control technique with staged reconstruction performed by experienced surgeons [4].

Although such guidelines exist for adult patients, the AAST did not include any recommendations for the pediatric population. Englum et al. reviewed 674 pediatric and adolescent patients who sustained blunt pancreatic trauma. When identifying predictors of operative management, higher grades of pancreatic injury and overall injury severity score were associated with operative management [1]. Mattix et al. specifically focused on pancreatic ductal injuries, and their results suggested that there may be an advantage to early operation in patients with ductal injuries [6]. Additionally, Beres et al. reviewed all pediatric pancreatic trauma from two Level I trauma centers, and patients with planned non-operative management had longer hospitalization, more total parenteral nutrition days, and more complications than patients managed operatively [7].

In our case, given the extent of injury sustained by the patient, a pancreaticoduodenectomy was the optimal surgical option. The initial damage control surgery was necessary, as the patient had sustained multiple other injuries that required immediate intervention, allowing a pancreaticobiliary surgeon to perform the pancreaticoduodenectomy in a controlled setting.

## Conclusion

Blunt abdominal trauma causing combined pancreatic and duodenal injury in the pediatric population is uncommon, and often requires surgery to stabilize the patient and identify the extent of the injuries. A consideration of our case is whether, in retrospect, the entire pancreas should have been resected to prevent future pancreatitis. Nevertheless, one would need to consider consequences of significant diabetes and complete lifelong insulin dependence. Our patient has only had mild cases of pancreatitis that were successfully treated conservatively. She also was found to have pathogenic variants in the CTR and SPINK1 genes, reportedly that combination which may cause hereditary predisposition to pancreatitis, which at the time of surgery was not known in her medical history. We believe a staged pancreaticoduodenectomy was the correct surgical decision despite the subsequent episodes of pancreatitis. Given the limited data available, this is a potential area for future investigation.

## Meeting presentations

No former presentations to disclose.

## CRediT authorship contribution statement

**Sarah E. Kim:** Project administration, Writing – original draft, Writing – review & editing. **Farah Al Rahmani:** Data curation, Writing – original draft, Writing – review & editing. **Rembrandt VanDruff:** Writing – original draft, Writing – review & editing. **Marc Mesleh:** Writing – review & editing. **J. Kayle Lee:** Supervision, Writing – original draft, Writing – review & editing.

## Declaration of competing interest

We have no conflicts of interest to disclose. All material is original, and it has not published elsewhere or submitted for another publication at this time. This current paper, if accepted, will not be published elsewhere in same/similar form without consent from the copyright holder.
